# Intracerebral Administration of *S*-Adenosylhomocysteine or *S*-Adenosylmethionine Attenuates the Increases in the Cortical Extracellular Levels of Dimethylarginines Without Affecting cGMP Level in Rats with Acute Liver Failure

**DOI:** 10.1007/s12640-016-9668-7

**Published:** 2016-09-08

**Authors:** Anna Czarnecka, Krzysztof Milewski, Radosław Jaźwiec, Magdalena Zielińska

**Affiliations:** 1 Department of Neurotoxicology, Mossakowski Medical Research Centre, Polish Academy of Sciences, 5 Pawińskiego Street, 02-106 Warsaw, Poland; 2 Mass Spectrometry Laboratory, Institute of Biochemistry and Biophysics, Polish Academy of Sciences, 5A Pawińskiego Street, 02-106 Warsaw, Poland

**Keywords:** Asymmetric dimethylarginine, Symmetric dimethylarginine, Hepatic encephalopathy, cGMP, *S*-Adenosylhomocysteine, *S*-Adenosylmethionine

## Abstract

Alterations in brain nitric oxide (NO)/cGMP synthesis contribute to the pathogenesis of hepatic encephalopathy (HE). An increased asymmetrically dimethylated derivative of l-arginine (ADMA), an endogenous inhibitor of NO synthases, was observed in plasma of HE patients and animal models. It is not clear whether changes in brain ADMA reflect its increased local synthesis therefore affecting NO/cGMP pathway, or are a consequence of its increased peripheral blood content. We measured extracellular concentration of ADMA and symmetrically dimethylated isoform (SDMA) in the prefrontal cortex of control and thioacetamide (TAA)-induced HE rats. A contribution of locally synthesized dimethylarginines (DMAs) in their extracellular level in the brain was studied after direct infusion of the inhibitor of DMAs synthesizing enzymes (PRMTs), *S*-adenosylhomocysteine (AdoHcy, 2 mM), or the methyl donor, *S*-adenosylmethionine (AdoMet, 2 mM), via a microdialysis probe. Next, we analyzed whether locally synthesized ADMA attains physiological significance by determination of extracellular cGMP. The expression of PRMT-1 was also examined. Concentration of ADMA and SDMA, detected by positive mode electrospray LC–DMS–MS/MS, was greatly enhanced in TAA rats and was decreased (by 30 %) after AdoHcy and AdoMet infusion. TAA-induced increase (by 40 %) in cGMP was unaffected after AdoHcy administration. The expression of PRMT-1 in TAA rat brain was unaltered. The results suggest that (i) the TAA-induced increase in extracellular DMAs may result from their effective synthesis in the brain, and (ii) the excess of extracellular ADMA does not translate into changes in the extracellular cGMP concentration and implicate a minor role in brain NO/cGMP pathway control.

## Introduction

Acute liver failure (ALF) is a clinical manifestation of sudden and severe hepatic injury resulting from a variety of causes (Felipo 2013). It may lead to impaired cerebral function known as hepatic encephalopathy (HE) due to accumulation of neurotoxic and neuroactive substances in the brain (Butterworth 2003). The pathophysiological basis of HE remains unclear; however, the generally accepted view is that toxic effects of ammonia on astrocytes play a key role (Albrecht and Jones 1999; Kosenko et al. 1997; Shawcross et al. 2007). One of the key aspects of ammonia toxicity is related to the alterations in brain nitric oxide (NO) signaling (Felipo 2013). Ammonia evokes activation of *N*-methyl-d-aspartate (NMDA) receptors and, in consequence, changes NO and cyclic GMP (cGMP) synthesis. It was repeatedly reported that this pathway is differently regulated and depended on the type of HE and/or its stage. In severe and acute HE, excessive brain glutamate release leads to the increased stimulation of the NMDA receptors resulting in NO release (Cauli et al. 2008) and cGMP accumulation (Hermenegildo et al. 2000; Hilgier et al. 2004). On the other hand, chronic HE is associated with the reduced cGMP synthesis (ElMlili et al. 2010).

Dimethylarginines (asymmetric, ADMA and symmetric, SDMA), l-arginine derivatives, are endogenous modulators of NO synthesis (Teerlink et al. 2009). ADMA is present in a high concentration in the brain and is a major endogenous NO synthases (NOSs) inhibitor and a competitive inhibitor of the cellular l-arginine (Arg) uptake through cationic amino-acid transporters (CAT) (Cooke 2000; Teerlink et al. 2009). SDMA may compete with Arg for uptake to the cell, thus limiting substrate availability for NOSs (Leiper and Vallance 1999). Dimethylarginines are formed by the degradation of methylated proteins (Cooke 2000; McDermott 1976) via protein arginine methyltransferases (PRMTs)-dependent transfer of the methyl group from *S*-adenosylmethionine (AdoMet) to Arg thus forming methylated Arg and *S*-adenosylhomocysteine (AdoHcy) that is subsequently hydrolyzed to homocysteine (Clarke 1993). PRMT-1 is the major enzyme that generates ADMA, whereas PRMT-2 synthesizes SDMA (Bełtowski and Kędra 2006). The major ADMA-eliminating route involves an enzymatic reaction catabolizing ADMA to citrulline and dimethylamine through ubiquitously localized dimethylarginine dimethylaminohydrolase (DDAH) (Nijveldt et al. 2003). Of interest, transamination of ADMA to d-keto-d-(*N*,*N*-dimethylguanidino) valeric acid by alanine-glyoxylate aminotransferase 2, distributed throughout the brain, is an additional metabolic pathway of ADMA removal (Abe et al. 2014).

A significant correlation between the increased plasma ADMA level and the degree of hepatic dysfunction has been found in patients with cirrhosis (Bajaj et al. 2013; Lluch et al. 2004, 2006; Richir et al. 2008), hepatitis C-related chronic liver disease (Vizzutti et al. 2007), severe acute alcoholic hepatitis (Mookerjee et al. 2007b), and ALF (Mookerjee et al. 2007a). The concentration of circulating ADMA seems to be regulated mainly by DDAH, which catalyzes its degradation (Leiper et al. 1999). Recently, Mookerjee (Mookerjee et al. 2015) reported a decreased expression of DDAH-1 isoform in the liver of cirrhotic patients. Moreover, in TAA-induced ALF in rodents, an increase in plasma ADMA level was shown to correlate with the reduced activity of DDAH in the liver (Bekpinar et al. 2015; Develi-Is et al. 2013) and brain tissue (Milewski et al. 2015). An increased ADMA content in plasma and brain tissue have also been reported in rat models of chronic cirrhosis (Balasubramaniyan et al. 2012; Laleman et al. 2005; Mookerjee et al. 2015).

A majority of the above studies have been focused on changes in peripheral tissues and/or in plasma. The contribution of dimethylarginines to the dysregulation of the NO/cGMP pathway in HE-affected brain remains obscure. In terms of neurological and cognitive impairment observed in HE patients, it is of interest to analyze in a well-established TAA rat model reproducing cerebral metabolic changes and symptoms of ALF, the synthesis of dimethylarginines directly in the brain. The extracellular space of the brain attained by microdialysis is the only compartment cohabited by blood-derived and brain-derived dimethylarginines and, as such, the only compartment in which relative contributions of the two pools can be assessed. Considering above, a novelty of the present study is a determination of the extracellular concentration of ADMA and SDMA in the brain of ALF rats. Physiological significance of locally synthesized dimethylarginines was analyzed by measurement of cGMP content extracellularly. Therefore, the aim of this study was to investigate: (i) extracellular brain concentration of dimethylarginines after infusion of the PRMT inhibitor *S*-adenosylhomocysteine or the methyl donor *S*-adenosylmethionine via a microdialysis probe; (ii) cGMP formation after *S*-adenosylhomocysteine infusion; and (iii) PRMT-1 expression in control and thioacetamide (TAA)-induced ALF rats.

## Materials and Methods

### Animals

The studies were conducted on male Sprague–Dawley rats of initial body weight between 250 and 300 g kept under standard laboratory conditions at room temperature (22 °C) under an artificial light/dark cycle (12/12 h), with free access to standard laboratory food and tape water. All procedures were conducted in accordance with the National Institutes of Health Guidelines for the Care and Use of Laboratory Animals and received a prior approval from the Bioethics Commission of the Academy, as compliant with Polish Law (of January 21, 2005). All efforts were made to reduce the number of animals and to minimize their suffering.

### Chemicals

ADMA, SDMA, ADMA-D6, thioacetamide, *S*-adenosylmethionine, and *S*-adenosylhomocysteine were provided by the Sigma-Aldrich Chemical Co. (Steinheim, Germany); isoflurane was from Baxter (Warsaw, Poland); HPLC grade ethanol (EtOH), HPLC grade formic acid, gradient grade acetonitrile (ACN), and LC/MS grade ACN from J.T. Baker (Deventer, The Netherlands) were purchased from Witko. MQ Water was purified with Millipore (Millipore, Bedford, MA, USA) MilliQ instrument.

### Acute Liver Failure Model

ALF with cerebral metabolic changes and symptoms typical of acute HE was induced by three i.p. injections of thioacetamide (TAA) (300 mg/kg b.w.) at 24-h intervals (Hilgier and Olson 1994; Hilgier et al. 1996) and sacrificed 24 h after third injection. Control rats received sodium saline solution (0.9 % NaCl).

### Microdialysis of the Rat Prefrontal Cortex

Bilateral microdialysis of the prefrontal cortex was carried out 24 h after the last TAA administration. The rats were anesthetized with 5 % isoflurane in air within 2 min and then maintained in anesthesia during the whole experiment with a 2 % isoflurane–air mixture. The body temperature was kept at 37.3 °C by a heating pad controlled by a rectal thermometer. The animals were fixed in a Stoelting stereotaxic frame. Briefly, concentric microdialysis probes (dialyzing membrane: diameter 0.5 mm; length 3 mm, CMA 12 Elite, CMA Microdialysis, Stockholm, Sweden) were implanted bilaterally into the prefrontal cerebral cortex through a small hole in the skull (stereotaxic coordinates according to the atlas of Paxinos and Watson (1986) were as follow: A/P + 3.0; l +(−) 1.0; D/V −3.5). The probes were perfused with artificial cerebrospinal fluid (aCSF), pH 7.4, containing: 126 mM NaCl, 2.4 mM KCl, 1.1 mM CaCl_2_, 0.8 mM MgCl_2_, and 0.5 mM KH_2_PO_4_ at a rate of 2.5 µl/min. Six fractions were collected every 40 min (0–240 min), starting 30 min after implantation of the probe. For stimulation, 2 mM *S*-adenosylhomocysteine (AdoHcy) or 2 mM, and methyl donor, *S*-adenosylmethionine (AdoMet) in a standard aCSF was infused commencing at 80 min, for 40 min, where after the medium was changed back to aCSF. A 2 mM concentration, as the lowest effective dose of the stimulants, was chosen after preliminary experiments conducted with 1, 2, and 5 mM concentrations. After completion of the microdialysis, the anesthetized rats were immediately sacrificed by decapitation. Dialysates were stored at −80 °C until further procedures were applied.

### Positive Mode Electrospray LC–DMS–MS/MS

The extracellular levels of ADMA and SDMA were analyzed using positive mode electrospray LC–DMS–MS/MS.

#### Sample Preparation

The sample (20 µl) was mixed with 80 µl of ADMA-D6 (IS) solution in EtOH (4 ng/ml). Solution was evaporated to dryness under nitrogen, reconstituted with 45 µl of ACN, and transferred to chromatographic vials.

Calibration curve was prepared using the same buffer as for microdialysis. Eight calibration points were prepared in the range from 0.33 to 40.7 ng/ml for ADMA and from 0.5 to 40 ng/ml for SDMA.

#### Sample Analysis

Samples were analyzed using Waters Xevo TQ-S triple quadrupole mass spectrometer coupled with Waters Acquity I-Class UPLC.

A 3-min HPLC method was set up on Waters HILIC equipped with 1.7 µm 2.1 × 100 mm column with thermostatic control at 70 °C. Mobile phase A was composed of 0.1 % FA in ACN, mobile phase B 0.1 % FA in MQ. Linear gradient from 20 to 70 % of phase B was used within 2.1 min with the flow rate of 0.65 ml/min. Injection volume was 3 μl. Retention time for both ADMA and SDMA was 0.81 min.

MS detector worked in ESI ionization in MRM mode. Separation was started with setting up MRM transitions that were highly specific for each compound. Using transitions 203.15 > 46.11 (collision energy 14) for ADMA and 203.15 > 172.17 (collision energy 12) for SDMA, we saw no crosstalk between signals of both compounds as it was previously reported in literature (Martens-Lobenhoffer et al. 2012; Zotti et al. 2008).

Monitored transmission for ADMA-D6 (IS) was 209.19 > 164.19 (collision energy 15).

MS parameters were capillary (kV) 3.00; cone (V) 30.00; source offset (V) 40.0; source temperature (°C) 150; desolvation temperature (°C) 550; cone gas flow (L/Hr-nitrogen) 150; desolvation gas flow (L/Hr-nitrogen) 1000; collision gas flow (ml/Min) 0.15; and Nebuliser gas flow (Bar) 7.00.

### Determination of cGMP in the Microdialysis

Tubes intended for cGMP analysis were coated with 5 µl of 4 mM EDTA. Samples of probes perfused with aCSF were collected every 40 min in the period of 80–240 min of perfusion, and 2 mM AdoHcy was infused for 40 min at 80 min, as indicated. Extracellular cGMP level was determined with cGMP Enzyme Immunoassay Biotrak (EIA) System (Amersham Biosciences) according to the manufacturer’s protocol with modifications (Hilgier et al. 2009).

### RNA Isolation and Real-Time PCR

Total RNA from the rat brain cortex was isolated using TRI Reagent (Sigma-Aldrich, St. Louis, MO, USA), and then 1 μg was reverse transcribed using the high-capacity cDNA reverse transcriptase kit (Applied Biosystems, USA). Real-time PCR was performed in 96-well plates with the ABI 7500 apparatus (Applied Biosystems, Warrington, UK) using the Applied Biosystems Taqman probe assay—PRMT-1 (Rn 00821202), *β*-actin (Rn 00667869). Each reaction contained 5 μl Taqman Universal PCR Mastermix in a total volume of 10, and 1 μl cDNA was added to the reaction. The real-time PCR reactions were performed for 10 min at 95 °C, followed by 40 cycles of 15 s at 95 °C, and 1 min at 60 °C. The results of the analysis were calculated in relation to the *β*-actin product, and results were presented according to an Equation (2^−ΔΔCt^) that gives the amount of target, normalized to an endogenous reference, and relative to a calibrator. C_t_ is the threshold cycle for target amplification (Livak and Schmittgen 2001).

### Protein Isolation and Western Blot Analysis

Approximately 50 mg of rat brain cortex tissue were homogenized in 5 volumes of Triton lysis buffer at 4 °C (20 mM Tris pH 6.8, 137 mM NaCl, 2 mM EDTA, 1 % Triton X-100, 0.5 mM dithiothreitol, and 1 mM phenylmethylsulfonyl fluoride) containing Protease inhibitor cocktail (Sigma-Aldrich, St. Louis, MO), and Phosphatase inhibitor cocktail (Sigma-Aldrich, St. Louis, MO, USA). The brain homogenate was centrifuged for 15 min at 12,000×*g*, 4 °C. The supernatants were used for total protein determination and Western blot analysis. Briefly, 30 μg of protein was boiled with gel sample buffer (Sigma-Aldrich, St. Louis, MO, USA), separated on sodium dodecyl sulfate–polyacrylamide gel electrophoresis and then transferred onto nitrocellulose membrane. Blots were blocked with 5 % nonfat dry milk in Tris-buffered saline Tween-20 buffer. Incubation with PRMT-1 (A33) antibody (1:1000, Cell Signaling, USA) was done in Tris-buffered saline Tween-20 buffer with 2.5 % nonfat dry milk at room temperature for 0.5 h followed by 10-min incubation with peroxidase-conjugated anti-rabbit antibodies (1:2500, Sigma-Aldrich, St. Louis, MO, USA) for detection by Clarity™ Western ECL Substrate (Bio-Rad, USA). The first antibody was stripped off with 0.1 M glycine and pH 2.9, and the second incubation (1 h, 20–22 °C) was performed with an anti-GAPDH antibody (1:5000, Sigma-Aldrich, Aldrich, St. Louis, MO, USA).

### Statistical Analysis

Biochemical parameters were analyzed by a repeated measures ANOVA with the post hoc Newman–Keuls test. Real-time PCR and Western blot results were analyzed by the Student’s *t* test. Values were expressed as the mean ± SEM or as a  % of control group. A significance level of *p* < 0.05 was considered to be statistically significant. All statistical analyses were performed using Statistica for Windows v. 8.0 (Statsoft. Inc., USA).

## Results

### The Extracellular Levels of Dimethylarginines (ADMA and SDMA)

**Fig. 1:**
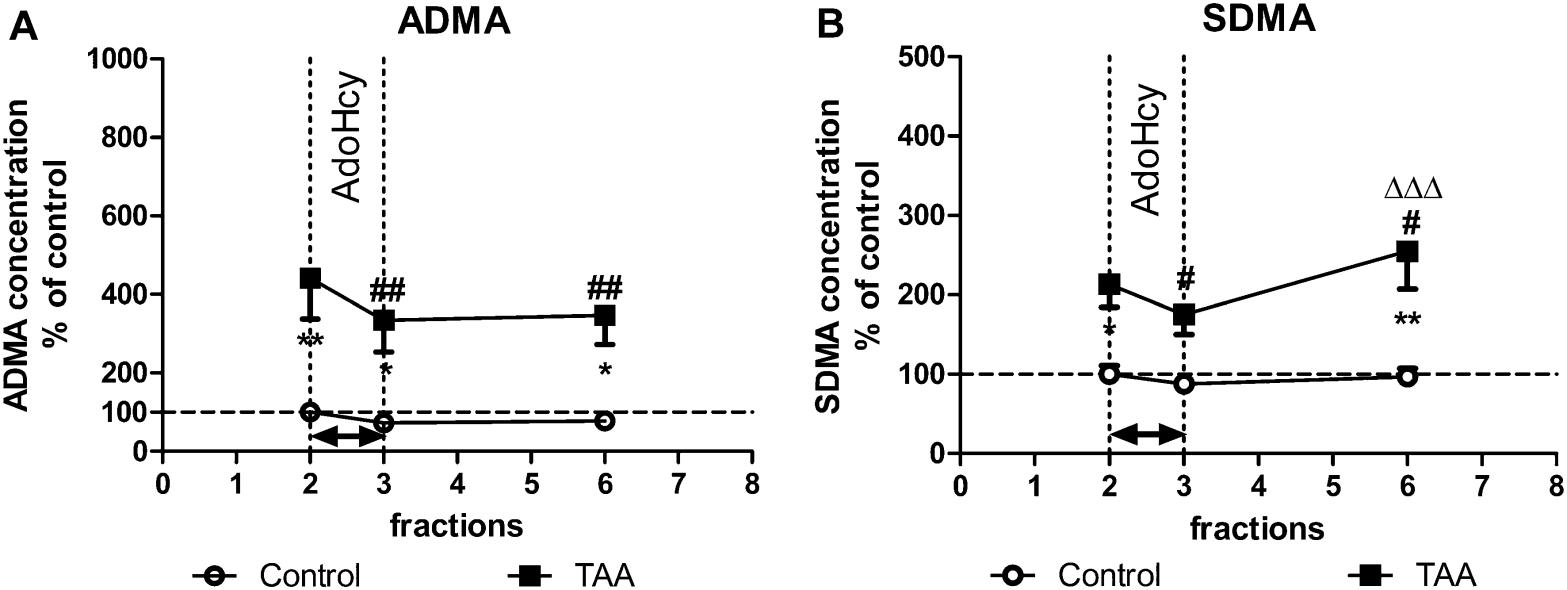
The extracellular levels of ADMA (**a**) and SDMA (**b**) in the prefrontal cortex of control and TAA rats: the effect of intracortical administration of exogenous AdoHcy (2 mM). The results are presented as  % of basal control level ± SEM, *n* = 6–7. *Symbols* indicate significance of differences in the post hoc Newman–Keuls test: *p < 0.05, **p < 0.01 versus control rats; ^*#*^ *p* < 0.05, ^*##*^ *p* < 0.01 versus fraction 2 of TAA rats; ^ΔΔΔ^ *p* < 0.001 versus fraction 3 of TAA rats

**Fig. 2:**
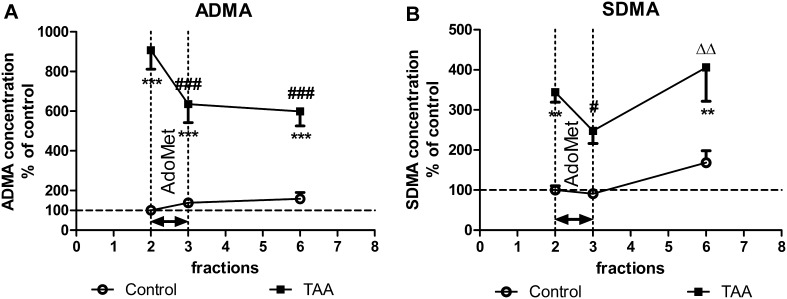
The extracellular levels of ADMA (**a**) and SDMA (**b**) in the prefrontal cortex of control and TAA rats: the effect of intracortical administration of exogenous AdoMet (2 mM). The results are presented as  % of basal control level ± SEM, *n* = 6. *Symbols* indicate significance of differences in the post hoc Newman–Keuls test: **p < 0.01, ***p < 0.001 versus. control rats; ^#^ *p* < 0.05, ^###^ *p* < 0.001 versus fraction 2 of TAA rats; ^ΔΔ^ *p* < 0.01 versus fraction 3 of TAA rats

The basal extracellular levels of ADMA and SDMA in the prefrontal cortex of control rats were 13.27 ± 1.77 and 9.17 ± 1.46 nM, respectively (Figs. 1, 2), and were maintained at this level during 240 min of the microdialysis (data not shown).

#### The Effect of 2 mM AdoHcy on ADMA and SDMA Concentration

The intracerebral infusion of AdoHcy via a microdialysis probe did not significantly affect ADMA concentration in control rats albeit a decreasing tendency could be observed (Fig. 1a). However, the TAA-evoked increase in ADMA concentration (to 450 %, *p* < 0.01) was reduced (by 25 %, *p* < 0.01) after AdoHcy infusion (Fig. 1a). The ANOVA for repeated measures revealed a significant effects of treatment, time, and a lack of treatment × time interaction (*F*_1,11_ = 13.54, *p* < 0.01; *F*_2,22_ = 8.57, *p* < 0.01, respectively).

On the other hand, the basal SDMA level was increased (to 200 %, *p* < 0.05) in TAA rats, whereas AdoHcy decreased it significantly (by 20 %, *p* < 0.05) (Fig. 1b). The ANOVA for repeated measures revealed a significant effects of treatment, time, and treatment × time interaction (*F*_1,11_ = 15.11, *p* < 0.01; *F*_2,22_ = 6.70, *p* < 0.01, *F*_2,22_ = 4.29, *p* < 0.05, respectively).

The AdoHcy-evoked decrease in ADMA level was still maintained in the 6th fraction (240 min of microdialysis, 2 h after stimulation), while at the same time point, the SDMA level returned to its basal value in TAA rats (Fig. 1b) which proves that observed decrease in dimethylarginines concentration resulted from AdoHcy infusion and not the nonspecific gradual reduction during time course of dialysis procedure.

#### The Effect of 2 mM AdoMet on ADMA and SDMA Concentration

Acute TAA administration resulted in a substantial increase in the basal extracellular levels of ADMA (to 900 % *p* < 0.001) when compared to control group. The intracerebral infusion of AdoMet resulted in a tendency toward an increase (by 30 %) in ADMA in control rats (Fig. 2a), whereas ADMA concentration in TAA rats was significantly decreased (by 30 %, *p* < 0.001). The ANOVA for repeated measures revealed significant effects of treatment, time, and treatment × time interaction (*F*_1,10_ = 51.48, *p* < 0.001; *F*_2,20_ = 8.57, *p* < 0.01; *F*_2,20_ = 17.02, *p* < 0.001, respectively). Significant interaction indicates that AdoMet differently modulates ADMA level in control and in TAA rats.

With regard to the symmetric methylated arginine derivative, the basal SDMA level was increased (to 350 %, *p* < 0.01) in TAA rats, whereas AdoMet decreased it significantly (by 30 %, *p* < 0.001) (Fig. 2b). There was no effect of stimulation in the control group. The ANOVA for repeated measures revealed a significant effect of treatment, time, and a lack of treatment × time interaction (*F*_1,10_ = 21.83, *p* < 0.001; *F*_2,20_ = 7.91, *p* < 0.001, respectively). Of note, the AdoMet-induced reduction in extracellular ADMA concentration was observed in TAA rats until the 6th fraction (240 min of microdialysis, 2 h after stimulation), while SDMA returned to the basal value (Fig. 2b).

### The Effect of the 2 mM AdoHcy on the Extracellular cGMP Level

**Fig. 3:**
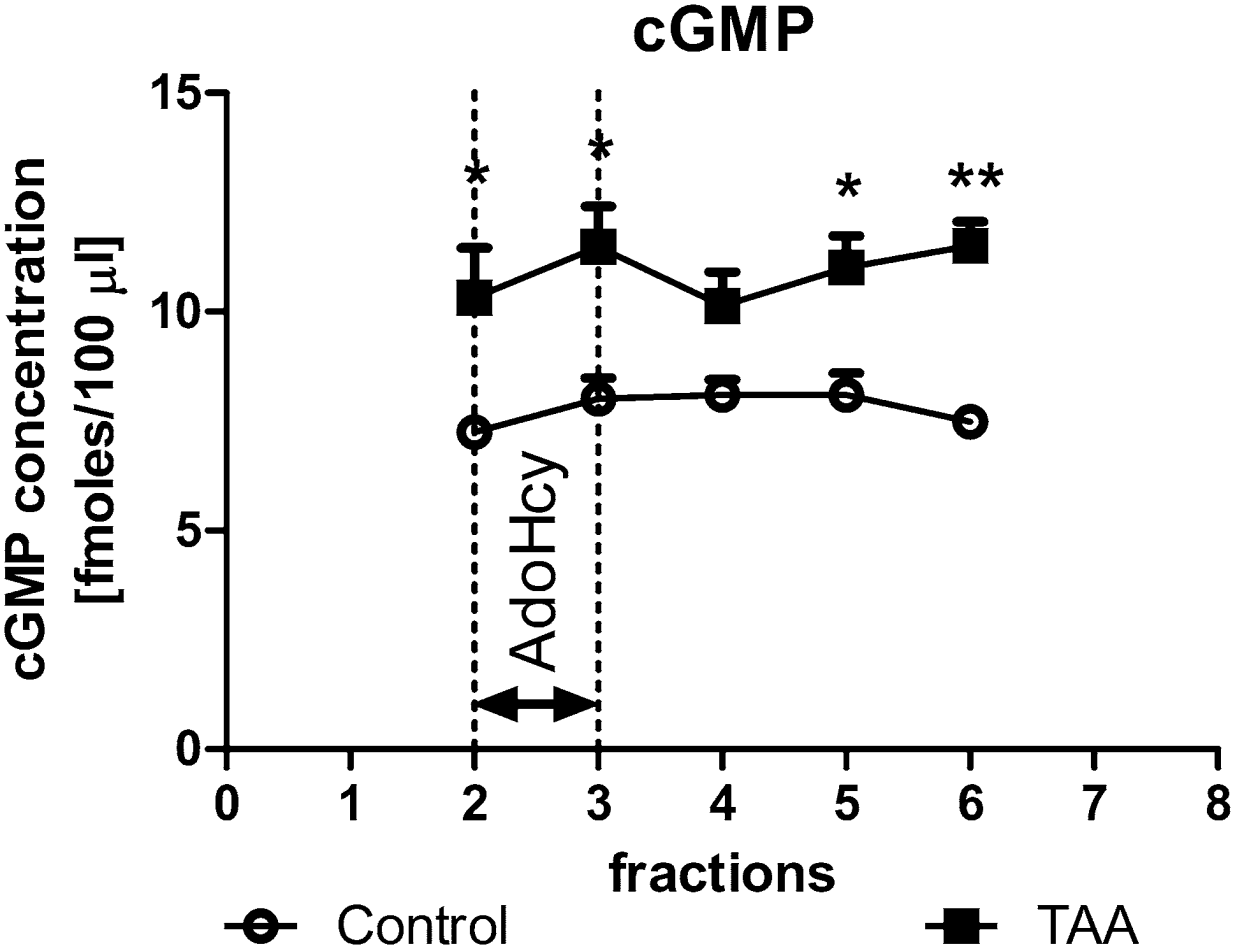
The extracellular levels of cGMP in the prefrontal cortex of control and TAA rats: effect of intracortical administration of exogenous AdoHcy (2 mM). The results are the mean ± SEM, *n* = 6–7. Symbols indicate significance of differences in the post hoc Newman–Keuls test: *p < 0.05, **p < 0.01 versus control rats

The basal extracellular concentration of cGMP in control rats was 7.25 ± 0.29 fmoles/100 µl (Fig. 3). TAA administration increased basal cGMP (to 142 %, *p* < 0.05) (Fig. 3). The ANOVA for repeated measures revealed a significant effect of treatment (*F*_1,11_ = 17.74, *p* < 0.01). There was no statistically significant effect of stimulation with AdoHcy.

### The Effect of ALF on PRMT-1 in the Rat Prefrontal Cortex

**Fig. 4:**
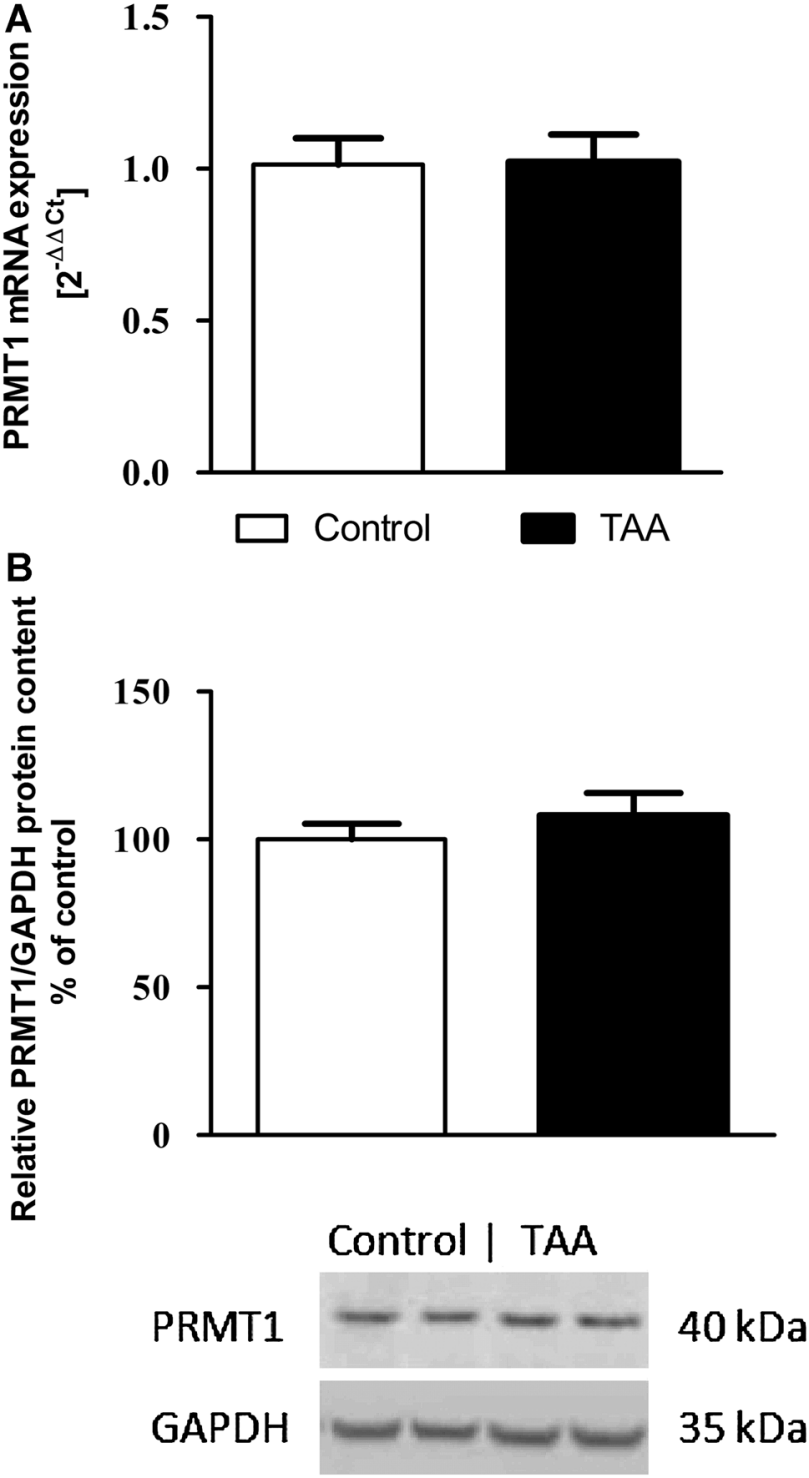
PRMT-1 mRNA (**a**) and protein (**b**) expression in the brain cortex of control and TAA rats. Results are presented as the mean ± SEM, *n* = 5–6

The PRMT-1 mRNA expression (Fig. 4a) and PRMT-1 protein level (Fig. 4b) in the brain cortex did not statistically significantly differ between TAA and control rats.

## Discussion

In the present study, we demonstrated an increase in the extracellular levels of dimethylarginines, ADMA, and SDMA in the prefrontal cortex of rats with TAA-induced ALF. The elevated ADMA concentration was not accompanied by a change in PRMT-1 expression. Intracerebral infusion of the PRMTs inhibitor AdoHcy or the methyl group donor AdoMet affected ADMA and SDMA synthesis in the rat brain. However, locally increased dimethylarginines simultaneously did not affect elevated extracellular level of cGMP in the prefrontal cortex of TAA rats.

ADMA plasma level is increased both in acute and chronic liver failure (see “Introduction” section). The study of Balasubramaniyan (2012) assessed an increased ADMA content in the brain homogenate of bile duct-ligated (BDL) rats using a semiquantitative method of immunoblotting. Recently, our group has reported that a moderate increase in ADMA brain tissue level correlated with decreased DDAH activity in the acute rat TAA model of HE (Milewski et al. 2015). The present study extends the above observations by demonstrating an increase in the extracellular levels of ADMA and SDMA in the brain cortex. It is worth noting that ADMA concentration in the cerebral tissue homogenate increased by 40 % (Milewski et al. 2015), whereas in the present study, extracellular level of ADMA was several times higher compared to control group. Presumably, the excess of ADMA is continuously released from the cell into the extracellular space. In the present study, we not analyzed an intracellular ADMA level that can be 10- to 20-fold higher than in plasma and reach ~5 μM (Teerlink et al. 2009). Such cellular ADMA concentration is highly above the IC_50_ for NOSs and might inhibit NO generation tonically, although this would depend also on the cellular concentration of arginine. To our knowledge, the extracellular content of both ADMA and SDMA in HE was not determined. ADMA is an endogenous competitive inhibitor of the neuronal NOS (nNOS) (Leiper and Vallance 2006) and a less potent inhibitor of its inducible isoform (Ueda et al. 2003). Therefore, the physiological concentration of ADMA is beneficial to neuronal cells due to inhibition of NO generation by nNOS and prevention of excitotoxic injury. The physiological concentration of ADMA is approximately 10-fold higher than that of other methylarginines, and therefore, ADMA seems to play a crucial role in controlling NO bioavailability (Blackwell 2010). Moreover, in vitro studies suggest that the ADMA synthesizing enzyme, PRMT-1 is more active in physiological conditions than the SDMA synthesizing enzyme, PRMT-2 (Lakowski and Frankel 2009), and dominant activity of PRMT-1 seems to keep the other PRMTs in check (Dhar et al. 2013).

In general, several mechanisms may lead to the accumulation of dimethylarginines: an increased methylation of proteins by PRMTs, augmented proteolysis and release of preformed methylarginines, impaired renal excretion, and (solely ADMA) impaired metabolism by DDAH (Bełtowski and Kędra 2006). Reduced DDAH protein expression and increased PRMT-1 were observed in alcoholic hepatitis livers (Mookerjee et al. 2007b), thus indicating that the increase in ADMA may result from both the decreased breakdown and/or increased production. However, the increased ADMA in plasma transported through the blood–brain barrier (BBB) may increase its brain level without any changes in brain dimethylarginine metabolism. Therefore, we were looking for a direct evidence that locally, brain metabolism of dimethylarginines is, at least partially, involved in ADMA/SDMA changes observed in HE. Using AdoHcy and AdoMet, we documented a local modulation of dimethylarginine synthesis, reflected by their extracellular level, both in control and TAA rats.

During methyl transfer, AdoMet is converted to AdoHcy, which can subsequently be hydrolyzed to adenosine and homocysteine through a reaction catalyzed by AdoHcy hydrolase (Palmer and Abeles 1979). In excess, AdoHcy is a potent competitive inhibitor of trans-methylation reaction (Finkelstein 1990; Mato et al. 1997) and may lead to decreased dimethylarginine level. Therefore, the experiments with 2 mM AdoHcy infused locally via microdialysis probe to the prefrontal cortex were performed. In line with our assumption, AdoHcy decreased both, ADMA and SDMA levels in TAA rats indicating effective modulation of brain dimethylarginine synthesis. In control rats, a tendency toward the decrease in ADMA level was observed. The competitive inhibition of PRMT reflected in decreased dimethylarginine level does not require translation of newly synthesized proteins therefore can be observed shortly after AdoHcy stimulation. Moreover, turnover of methylated proteins occurs at a high rate (Teerlink 2005) thus formation of dimethylarginines should be considered as a constant and fast process.

Direct infusion of AdoMet to the brain cortex of TAA rats decreased extracellular concentration of dimethylarginines. The possible explanation of this phenomenon is associated with a feedback inhibition of PRMT by AdoHcy, formed from AdoMet upon trans-methylation (Gibson et al. 1961). The intensity of this inhibition was reflected by the diminished dimethylarginine levels, which were also reduced after AdoHcy administration, what supports the above explanation. Intracortical infusion of AdoMet to TAA rat brain, in conditions of insufficient amount of the substrate (Huang et al. 1999), may lead to intensive methylation of many acceptors of methyl group (Fontecave et al. 2004; Lu 2000) and formation of AdoHcy (Finkelstein 1990; Mato et al. 1997). Therefore, the removal of AdoHcy is critical step. AdoHcy is converted to homocysteine and adenosine in a reversible reaction catalyzed by AdoHcy hydrolase, and hydrolysis occurs only after rapid product removal (Finkelstein 1990; Mato et al. 1997). Therefore, a strong feedback inhibition observed exclusively in TAA rats suggests that these processes are impaired in HE. A noticeable increase (~30 %) of AdoHcy level in the brain cortex tissue of TAA rats confirmed this assumption (manuscript in preparation). The hypothesis is also supported by the observation that in control rats AdoMet did not reduce the levels of dimethylarginines. In the present study, a significant interaction between time and treatment indicates an opposite modulation of ADMA level in control and TAA rats by AdoMet. Similarly, AdoMet was effective only in TAA rats, significantly reducing (by ~30 %) extracellular SDMA level increased by TAA.

On the other hand, the possibility that other nonspecific effects of AdoMet besides the inhibition of PRMT activity by excess of AdoHcy may contribute to the diminished dimethylarginine levels cannot be excluded. AdoHcy apart of inhibiting trans-methylation reaction may promote inhibition of DDAH activity after its conversion to homocysteine (Lentz et al. 2003) contributing to ADMA accumulation. However, in our experiments such effect was not observed. Summing up, the feedback inhibition of PRMT enzyme by AdoHcy seems the most reliable explanation of the observed decrease in ADMA concentration.

In our study, the expression of PRMT-1 in the brain cortex of TAA rats is not altered. However, PRMT-1 protein expression measured by immunoblotting does not reflect an actual activity of this enzyme, which is regulated by a number of factors, like PRMT-binding proteins or posttranslational modifications (automethylation, phosphorylation, and deimination) (Bedford and Clarke 2009). Therefore, a more comprehensive study would be of interest to provide a reliable conclusion regarding dimethylarginine synthesizing enzyme under ALF conditions.

It can be assumed that transport of dimethylarginines through the BBB contributes to the observed increase in their extracellular level in TAA rat brain. However, an immediate 30 % reduction of ADMA and SDMA level after AdoHcy or AdoMet infusion proves the hypothesis that an increased brain content of dimethylarginines resulted from the synthesis in the brain. More effective decrease in dimethylarginine levels after AdoHcy and feedback inhibition of their synthesis after AdoMet observed only in TAA rats may indicate an increased activity of PRMT enzymes in ALF. Furthermore, it should be noticed that our study supports the view that PRMT-1 is the dominant enzyme among other methyltransferases (Dhar et al. 2013). TAA administration evoked a more pronounced increase in ADMA (4- to 9-fold) than SDMA (2.5- to 3-fold) level. Moreover, decrease in the extracellular SDMA level after AdoHcy was lower than that of ADMA, furthermore, the effect of AdoHcy or AdoMet on SDMA persisted for a shorter period of time. This observation indicates presumably different activity and inhibitor affinity of PRMT-1 and PRMT-2 enzymes what is reflected by distinct contribution of ADMA and SDMA to the pathogenesis of HE.

In our study, the concentration of cGMP was measured in the microdialysis, not in the tissue. However, since diffusion of this molecule to the extracellular space is an instant process, the obtained data do genuinely reflect the activity of the NO/cGMP pathway in the brain tissue (Fedele and Raiteri 1999). We investigated the physiological significance of changes in cellular ADMA level evoked by AdoHcy on the extracellular cGMP concentration. In contrast to AdoMet, a methyl donor, a nonspecific reaction of methylation, is unlikely to occur (Fontecave et al. 2004; Lu and Mato 2012). The basal extracellular cGMP level was increased in the TAA rat cortex in line with earlier results (Hermenegildo et al. 2000; Hilgier et al. 2004) demonstrating the activation of NO/cGMP pathway in acute stage of HE. Why in our experimental setting AdoHcy did not affect cGMP synthesis, despite the persistent decrease in the extracellular ADMA level, seems to stay unsolved. It should be repeated that ADMA plasma and/or brain level is increased both in acute and chronic liver failure (see “Introduction” section). In contrast, NO/cGMP pathway is differently altered, depending on HE severity (Felipo 2013). Therefore, translation of changes in dimethylarginine contents into cGMP level may not be direct. In the present study, for the first time, we manipulated with the synthesis of dimethylarginines and investigated its influence on cGMP content locally in the brain of ALF rats. Other reports suggesting a direct link, between increased plasma ADMA concentration and cognitive impairment in TIPS patients or BDL rat model of HE (Bajaj et al. 2013; Huang et al. 2010) relied on a correlative assumption. Herein, we suggest that association between extracellular ADMA and cGMP contents is unlikely, especially in the studied model of ALF, when NO release is to a greater extent controlled by excitotoxic mechanisms resulting from overactivation of NMDA receptors.

Moreover, other possibilities of cerebral and/or systemic consequences of elevated ADMA such as influencing brain vascular constriction leading to the cerebral blood flow (CBF) dysregulation, oxidative stress, and inflammation should be considered. To this date, only circumstantial and correlative evidence for a role of ADMA as a mediator of selected processes in HE are available. Increased circulating ADMA levels, which inhibits NO synthesis, may be associated primarily with endothelial dysfunction that somehow can be translated on changes of CBF considered as a causative and a predictive factor of overt HE. However, the exact mechanism by which direct effects of ADMA in the brain are translated into CBF changes during HE has not been elucidated in detail.

Summing up, our study demonstrates an increase of the extracellular levels of dimethylarginines in the prefrontal cortex of TAA rats. The obtained results prove that brain synthesis of ADMA and SDMA is an effective process and is altered under conditions of ALF. Moreover, the TAA-induced increase in the extracellular cGMP level is not directly affected by local modulation of dimethylarginine synthesis.
